# FamuPlus – a pilot project to support self-oriented learning through self-created learning outcomes in clinical electives

**DOI:** 10.3205/zma001870

**Published:** 2026-09-15

**Authors:** Andrea Punz, Thomas Breakell, Thomas Kühlein, Sigrid Harendza, Raphael Kunisch, Bettina Engel

**Affiliations:** 1 Friedrich-Alexander-Universität Erlangen-Nürnberg, Uniklinikum Erlangen, Institute of General Practice, Erlangen, Germany; 2 University Medical Center Hamburg-Eppendorf, III. Medical Clinic, Hamburg, Germany; 3 University of Augsburg, Faculty of Medicine, Department of General Practice, Augsburg, Germany; 4 Friedrich-Alexander-Universität Erlangen-Nürnberg, Medical Campus Upper Franconia, Institute of General Practice, Bayreuth, Germany

**Keywords:** self-oriented learning, clinical electives, learning outcomes, SMART criteria, medical education, project implementation

## Abstract

**Background:**

Clinical electives in German medical education provide valuable insights into healthcare practice but often lack structured learning guidance. The FamuPlus project was developed to support medical students in defining and pursuing self-created learning outcomes to foster self-oriented learning (SOL), which is recognized as a key competence for lifelong learning and professional development in medical practice. In this study, self-created learning outcomes describe a guided process.

**Project description:**

Within FamuPlus, seven students completed a brief online training introducing the SMART (specific, measurable, achievable, relevant, and time-bound) framework and the learning domains of Bloom’s taxonomy. Thereafter, participants formulated individual learning outcomes prior to their clinical elective and reviewed them with their clinical teachers (e.g. general practitioner or ward physician, etc.) at the beginning of their independently chosen, monthlong, clinical elective on site. Throughout the elective, students reflected on their progress using an online self-evaluation tool and engaged in guided follow-up discussions about their experiences. To evaluate the intervention, a control group was additionally surveyed at the end of the clinical elective.

**Results and experiences:**

Students reported that defining their own learning outcomes improved focus, motivation, and perceived learning success it also seemed to promote self-efficacy during their electives. However, designing measurable learning outcomes was challenging.

**Discussion and conclusion:**

FamuPlus appears to be a feasible approach to support medical students in strengthening SOL during clinical electives. Despite the small, potentially highly motivated cohort the findings suggest beneficial effects of FamuPlus on perceived learning success, motivation and focus. Refinement and broader implementation are warranted to strengthen learner-centred education in clinical electives. The low-threshold online format and minimal faculty workload make the approach transferable to other faculties. For widespread implementation, careful consideration should be given to the involvement of faculty staff, depending on the available resources as adequate infrastructure is essential for sustainable implementation.

## Zusammenfassung

**Hintergrund:**

Famulaturen ermöglichen im deutschen Medizinstudium wertvolle Einblicke in die klinische Praxis, gehen jedoch häufig nicht mit einer strukturierten Lernbegleitung einher. Vor diesem Hintergrund wurde das Projekt FamuPlus entwickelt, um Medizinstudierende bei der Formulierung und Umsetzung individueller Lernergebnisse zu unterstützen und dadurch selbstorientiertes Lernen (SOL) zu fördern. SOL gilt als eine zentrale Kompetenz für lebenslanges Lernen sowie die kontinuierliche berufliche Weiterentwicklung im ärztlichen Kontext. Die im Rahmen dieser Studie formulierten individuellen Lernergebnisse entstanden dabei innerhalb eines gezielt angeleiteten Prozesses.

**Projektbeschreibung:**

Im Rahmen von FamuPlus absolvierten sieben Studierende ein Online-Training, welches in das SMART-Prinzip (spezifisch, messbar, erreichbar, relevant und zeitgebunden) sowie in die Lernzieldomänen der Bloom’schen Taxonomie einführte. Anschließend formulierten die Teilnehmenden vor Beginn ihrer Famulatur individuelle Lernergebnisse, die sie zu Beginn ihrer selbstständig gewählten, vierwöchigen Famulatur gemeinsam mit den betreuenden Lehrpersonen vor Ort (z. B. Hausärzt:innen oder Stationsärzt:innen) besprachen. Während der Famulatur reflektierten die Studierenden ihren Lernfortschritt mithilfe eines Onlinetools und teilten im Rahmen von moderierten Fokusgruppen-Interviews ihre Erfahrungen. Zur Evaluation der Intervention wurde zusätzlich eine Kontrollgruppe am Ende ihrer Famulatur im Rahmen von Fokusgruppen befragt.

**Ergebnisse und Erkenntnisse:**

Die Studierenden berichteten, dass die Festlegung eigener Lernergebnisse ihren Fokus, Motivation und das empfundene Lernerfolgserlebnis verbessert habe. Zudem schien dies ihre Selbstwirksamkeit während der Famulaturen zu fördern. Die Formulierung messbarer Lernergebnisse stellte jedoch eine Herausforderung für die Studierenden dar.

**Diskussion und Zusammenfassung:**

FamuPlus stellt einen vielversprechenden Ansatz dar, um Medizinstudierende während ihrer Famulaturen gezielt bei der Entwicklung selbst-orientierten Lernens (SOL) zu unterstützen. Trotz der kleinen und potentiell hoch motivierten Stichprobe weisen die Ergebnisse auf positive Effekte hinsichtlich des subjektiven Lernerfolgs, der Motivation und der Fokussierung hin. Eine Weiterentwicklung sowie eine breitere Implementierung erscheinen daher sinnvoll, um die Lernendenzentrierung in Famulaturen weiter zu stärken. Das niedrigschwellige Online-Format und der geringe zusätzliche Aufwand für Lehrende begünstigen eine Übertragbarkeit auf andere Fakultäten. Für eine umfassende Einführung sollte jedoch die Einbindung des Lehrpersonals sorgfältig an die verfügbaren Ressourcen angepasst werden, da eine angemessene infrastrukturelle Unterstützung für eine nachhaltige Umsetzung unerlässlich ist.

## 1. Introduction

Clinical electives are an integral part of undergraduate medical education in Germany, designed to provide students with first-hand experience in both inpatient and outpatient healthcare settings. During the clinical study phase (third to fifth year of undergrade curriculum),students are required to complete four, month-long electives in disciplines of their choice [https://www.gesetze-im-internet.de/_appro_2002/BJNR240500002.html]. While the formal structure of these electives is well defined regarding duration and organizational aspects, there are no content-related standards or required learning outcomes. Consequently, students’ learning experiences often depend heavily on individual initiative and the local teaching environment.

At the same time, the complexity of modern healthcare demands that medical students acquire not only knowledge and skills but also the capability of self-oriented learning (SOL) – a concept emphasizing learners’ responsibility for planning, monitoring, and evaluating their own learning process [1]. SOL is recognized as a key competence for lifelong learning and professional development in medical practice [2], [3], [4], [5], [6]. Self-oriented, self-regulated and self-directed learning are closely related constructs that describe different dimensions of learner autonomy within educational research. Although these concepts overlap in their emphasis on learner agency, they differ in scope: self-oriented learning foregrounds personal meaning and orientation, self-regulated learning highlights strategic regulation of learning processes, and self-directed learning addresses responsibility for organizing and managing one’s learning trajectory. In this paper the term SOL means to emphasize the importance of familiarizing medical students with a concept that promotes lifelong learning as a medical doctor [1]. The explicit use of learning outcomes stimulate vital parts of SOL, such as reflection, planning and self-efficacy. They provide explicit statements of what learners aim to achieve and help align expectations between students and supervisors [7], [8]. By defining personal learning outcomes, students are encouraged to take ownership of their learning, focus their efforts, and monitor progress more effectively. Previous studies have shown that clarity regarding learning objectives enhances both motivation and teaching engagement during clinical training [9]. 

A structured approach to formulate individual learning outcomes offer the SMART framework and Bloom’s taxonomy. The SMART framework is a widely used model for defining effective and measurable objectives. The acronym stands for specific, measurable, achievable (or attainable), relevant, and time-bound. It provides a structured approach to formulating learning goals, ensuring that objectives are clearly defined and practically attainable [10]. Bloom’s Taxonomy of Learning Domains provides a comprehensive framework for classifying educational objectives across three domains of learning: the cognitive, affective, and psychomotor. Together, these domains emphasize that effective learning encompasses intellectual, emotional, and physical dimensions. The taxonomy remains a foundational model for designing holistic educational objectives and assessments [11], [12]. 

To support students in developing such learning strategies, the FamuPlus project was created at Friedrich-Alexander-Universität Erlangen-Nürnberg. The underlying idea in developing FamuPlus was to preserve the inherently exploratory nature of clinical electives while simultaneously providing guidance for self-oriented learning. Designing a project that fosters SOL by enabling students to develop their own learning outcomes seemed to be a valuable approach. 

The purpose of this project report is to describe the development and implementation of FamuPlus, share the experiences of participating students, and discuss lessons learned from the pilot phase regarding its potential to enhance SOL in clinical electives.

## 2. Project description

The FamuPlus project was developed at Friedrich-Alexander-Universität Erlangen-Nürnberg to support medical students in designing and pursuing individual learning outcomes during their clinical electives. The overall aim was to strengthen self-oriented learning (SOL) by providing students with a structured yet flexible framework for goal formulation, reflection, and feedback.

### 2.1. Project design and implementation

FamuPlus was designed as a pilot project and implemented in a small cohort of medical students during their clinical electives between summer and winter term of 2021. Participation was voluntary. Ethical approval was obtained from the Ethics Committee of Friedrich-Alexander-Universität Erlangen-Nürnberg. Written informed consent was collected from all participants, and confidentiality was maintained throughout the project.

The development of the project was implemented by a physician and a medical student. There were no participants or other clinical teachers involved in the development. 

A total of 14 medical students from Friedrich-Alexander-Universität Erlangen-Nürnberg participated in the pilot phase of FamuPlus. Seven students took part in the intervention group (IG) and completed their electives with FamuPlus guidance, while seven others completed their (independently chosen and organized) electives without the project (control group, CG). Students from both groups were recruited through an open call (at different timings) for participation and therefore from different semesters. The intervention group included five female and two male students who volunteered as part of a general practice-oriented mentoring program in Bavaria. The elective fields selected by the IG included general medicine, palliative care, internal medicine, and general surgery. In the CG, the chosen specialties were nuclear medicine, psychiatry, ambulatory anaesthesia, general medicine, internal medicine, and vascular surgery. The CG group was added to the pilot project to be able to observe first effects of the intervention. The project followed a stepwise schedule that accompanied students before, during, and after their elective period (see figure 1 (Fig. 1)).

#### 2.1.1. Step 1: Preparation phase

Before starting their elective, students completed a short online training introducing principles of learning theory, the SMART framework for goal formulation, and Bloom’s taxonomy of learning domains (cognitive, affective, and psychomotor). Videos were created for the training, by the developing physician, to introduce the concept, importance, and structure of learning objectives. This training aimed to equip students with the necessary tools to formulate meaningful and achievable learning outcomes. To support the process, a digital SMART template that is shown in table 1 (Tab. 1) including examples was provided. It guided students through defining each learning outcome according to the SMART criteria and assigning it to one of Bloom’s domains.

#### 2.1.2. Step 2: Planning and clinical teaching

After completing the training, students formulated individual learning outcomes for their upcoming elective. These were shared with their clinical teachers via email from the FamuPlus team. While not part of the FamuPlus team, clinical teachers were encouraged to discuss the goals with the students at the beginning of the placement to ensure a shared understanding of learning expectations.

#### 2.1.3. Step 3: Clinical phase

Throughout the elective, students completed a weekly online self-evaluation form that contained their predefined learning objectives. For each objective, they rated their current level of achievement on visual scale ranging from zero to a hundred percent (including a T0 rating, before the start of their elective). Ratings from the previous week were not displayed in order to reduce potential bias in self-assessment. The continuous scale was selected to further reduce potential bias in self-assessment by limiting anchoring effects. In the middle of the elective, participants were invited to an informal interview about their experiences so far, using a semi-structured discussion guide (see attachment 1 ). This reflection encouraged students to adjust their focus if needed and to become more aware of their own learning strategies. The seven interviews were conducted by AP via synchronous video calls.

#### 2.1.4. Step 4: Feedback and reflection

At the end of the clinical elective, all participants took part in online focus group discussions based on a semi-structured guideline (see attachment 2 ) to reflect on their experiences with FamuPlus. 

### 2.2. Data collection and evaluation

The implementation of the pilot project was monitored through qualitative research to better understand its educational impact and feasibility. The focus group discussions and the specifically developed semi-structured interview guide were therefore used for reflection and to explore the potential effect of FamuPlus on SOL. Four focus group discussions were conducted (two per IG and CG, one group with three and one with four participants). The clinical teachers did not participate in these sessions. Each discussion lasted approximately 90 minutes and was facilitated by the same physician-medical student team following the structured discussion guide. Focus group transcripts and interview data were analysed using Kuckartz’s approach to qualitative content analysis [13]. Pre-existing categories related to SOL in clinical electives (people, learning, and circumstances) were used as guiding themes [14], and additional (sub-)categories were developed inductively during the coding process (see table 2 (Tab. 2)). The project team, consisting of medical student and teaching physician, collaboratively discussed and refined the coding structure to ensure consistency. When differences arose in code assignment, the researchers discussed the relevant parts of the transcripts and codes and agreed on compromises. Any disagreements were resolved within the research team without the need to consult other researchers. Moreover, the student designed learning outcomes were collected and analysed according to the SMART approach.

## 3. Results and experiences

### 3.1. Development of learning outcomes

The seven participants in the FamuPlus group formulated a total of 29 individual learning outcomes, ranging from three to five per student. Remarkably, the students of the CG did not design any learning outcomes. According to Bloom’s taxonomy, 18 of the learning outcomes were related to psychomotor skills, six to cognitive knowledge, and five to affective attitudes (see table 3 (Tab. 3)). Students reported in the focus group discussions that it was sometimes challenging to formulate outcomes that were both measurable and realistic according to SMART criteria. Nevertheless, the process of defining them was described as valuable and motivating.

### 3.2. Students’ experiences and perceived effects

Students who participated in FamuPlus described a clear improvement in their focus, motivation, and sense of self-efficacy. They felt more proactive in seeking learning opportunities, communicating their interests, and requesting feedback. Several participants emphasized that articulating their learning goals helped them to better recognize their progress and realistically assess what could be achieved during a one-month clinical elective.

*“It was a good guideline through the clinical elective.” (IG)*

*“Having my learning outcomes in mind helped me to stay focused and to realize what was realistic [to be achieved].” (IG)*

*“Above all it helped me to set focus – because everything was new and you could learn so many different things, and that [the concept of learning outcomes] really helped me to focus on something to learn thoroughly. And I guess I would do that again for my next clinical elective. (IG)*

Weekly self-evaluations were considered useful for reflection and self-monitoring. Students noted that the process encouraged them to take initiative, even in busy clinical environments.

*“I asked my supervisor, “These are my goals – what else could I learn here?”” (IG)*

In contrast, students in the control group who had not participated in FamuPlus reported uncertainty about how to define or prioritize their learning during electives. Several expressed that the idea of pre-defining and discussing learning outcomes with clinical teachers would have been helpful for their future placements.

*“I realized during this discussion that it would be beneficial to design and communicate learning outcomes before my next elective.” (CG)*

### 3.3. Perceived impact on clinical teaching

Students observed that FamuPlus positively influenced communication with their clinical teachers. They seemed to become more aware of students’ individual learning goals and responded more deliberately to support them. Moreover, all participants in the intervention group reported having an appointed clinical teacher, while only three of the seven control students did.

*“My clinical teacher sometimes said, “You mentioned you wanted to learn this – let’s try it now.” That was very helpful.” (IG)*

### 3.4. Identified challenges and limitations of learning goals

Some participants found it difficult to develop measurable goals, particularly when addressing affective or attitudinal learning outcomes. A few students initially felt that empathy or patient-centred attitudes could not be explicitly “learned,” while others found such outcomes to be essential reminders of the human aspect of clinical care.

*“I found it very useful to have learning outcomes, but also difficult to quantify them.” (IG)*

*“(…) I do think [formulating learning goals is] something that can be learned, but probably over time. I think it’s more a matter of experience. I’m not sure a doctor could really teach me [patient centred attitude and empathy] directly; it’s probably something that happens more intuitively, through experience.” (IG)*

*“[…] I’m someone who […] tends to include […] interpersonal aspects intuitively and reflects on them a bit […]. But I think it’s really great to actually write them down and consciously think about them.” (IG)*

## 4. Discussion

The FamuPlus project was designed to provide a structured yet flexible framework for SOL during clinical electives. The pilot phase demonstrated that supporting students in formulating and reflecting on their own learning outcomes can strengthen their autonomy, focus, and engagement in clinical training. Students who participated in FamuPlus described a greater sense of responsibility for their learning and felt more empowered to request meaningful learning opportunities. This aligns with previous findings showing that clearly defined learning goals can enhance motivation, focus, and teaching quality in the clinical environment [2], [9]. By introducing structured learning outcome development as a preparatory activity, FamuPlus encouraged students to enter their electives with a more deliberate learning plan and to evaluate their own progress. A key lesson from the project was that even minimal structural support – such as a short online module and a simple template for designing learning outcomes – can make a meaningful difference. Students seemed to value the process of developing learning outcomes as much as achieving them. The act of reflecting on their learning goals created an ongoing dialogue with clinical teachers and helped students articulate their learning needs more confidently.

However, several challenges became apparent. Many participants struggled to formulate measurable or time-bound outcomes according to the SMART framework, particularly when dealing with affective learning outcomes (e.g., empathy, patient communication). This difficulty has also been described in previous research [10], [15]. Defining measurable learning outcomes enables students to evaluate and reflect on their own progress. Nevertheless, it may indicate that while SMART criteria provide a helpful structure, they are not universally applicable to all domains of medical learning, especially those involving professional attitudes or interpersonal skills. Consequently, the online module should place greater emphasis on illustrating how such complex or affective goals can be meaningfully defined and operationalized – using concrete examples, reflective prompts, or adapted frameworks – as suggested by recent educational literature [16], [17].

Another important insight of the FamuPlus pilot project relates to the relationship between students and their clinical teachers. Students reported that their assigned clinical teachers became more attentive to their learning needs when goals were explicitly communicated. This supports earlier findings suggesting that students’ clarity about learning outcomes can positively influence supervisors’ engagement and teaching behaviour [9]. Thus, FamuPlus may serve not only as a student support tool but also as a low-threshold faculty development measure by sensitizing teachers to students’ individual needs. It would therefore be valuable to consider involving clinical teachers into (e.g. separate) focus group discussions in future iterations of the project.

However, beyond the influence of teacher-student relationships, an important limitation of this pilot project must be acknowledged: The intervention comprised several components that may have affected SOL in different ways. Yet, due to the nature of its implementation, these elements were not evaluated separately. Exploring this aspect could provide valuable insights and should be considered in future research.

While the qualitative feedback was consistently positive, the intervention group likely included particularly motivated students who voluntarily participated in a general practice-oriented mentoring program. Therefore, the observed effects should be interpreted with caution and may not fully generalize to all medical students. Future iterations should include a broader and more diverse participant group to validate these experiences and explore long-term effects on self-oriented learning. Another aspect to be highlighted is that students designing their own learning outcomes may lead to incomplete curricular coverage. There is currently no curriculum for clinical electives in German medical schools, but it may be necessary to add some degree of external guidance through a overarching approach in the future. Finally, the feasibility of the online-based approach deserves attention. Given the growing number of medical students, scalable and resource-efficient interventions are needed. Previous studies suggest that online training can be as effective as face-to-face instruction in undergraduate medical education [18]. The FamuPlus experience supports this notion, indicating that online components can successfully introduce key learning concepts without additional faculty workload. Further insights gained from the pilot project related to the practical demands of its design. Particularly the workload associated with conducting focus group discussions and the technical integration of student-generated learning outcomes into a digital platform for weekly evaluation. Given the small sample size, learning objectives were transferred manually; however, this approach would result in an unsustainable workload if implemented on a larger scale. Future iterations of the project will therefore streamline these processes by enabling students to formulate and document their learning objectives directly within a university-based online platform. There, progress can be monitored within the same digital interface. In addition, opportunities for peer exchange without formal facilitation (replacing focus group discussions) will be incorporated to reduce organisational burden while preserving reflective dialogue.

Overall, the project highlights the potential of integrating structured, student-defined learning outcomes into the flexible framework of clinical electives. This approach encourages active engagement, fosters reflection, and strengthens the teacher-student relationship – key components of SOL and lifelong professional development. The FamuPlus experience was perceived as a motivating and valuable addition to the elective period, with students reporting a greater sense of ownership over their learning process, a clearer structure, and improved reflection on their personal progress.

## 5. Conclusion

The FamuPlus project showed that supporting medical students in formulating and reflecting on their own learning intended outcomes can enhance SOL during clinical electives. Combining brief preparatory training with structured goal development and discussions with their clinical teachers helped students become more focused and confident in shaping their learning experiences, while also increasing teachers’ awareness of individual learning needs. Although defining measurable goals – especially in the affective domain – remains challenging, FamuPlus can be a feasible and motivating approach for both students and educators. Despite the fact that the pilot project included a small cohort of students of allegedly highly motivated students and the different components of the project need further, separated evaluation indicates positive effects on medical students’ SOL. Future project phases will build on these insights by refining training materials, expanding participation to larger student cohorts, and exploring how similar goal-oriented frameworks can be implemented across other stages of medical education. In doing so, FamuPlus may be able to contribute to the ongoing development of structured, learner-centred approaches in clinical training and supports the cultivation of lifelong learning skills essential for modern medical practice.

## Notes

### Ethics approval and consent to participate

Ethical approval for this project was obtained from the Ethics Committee of Friedrich-Alexander-Universität Erlangen-Nürnberg (22-28-S). Written informed consent was obtained from all participants prior to participation. All data was anonymized and handled in accordance with institutional and ethical standards.

### Availability of data and materials

The qualitative data generated and analysed during this project is available from the corresponding author upon reasonable request. Due to the sensitive nature of focus group transcripts, full data cannot be made publicly available to protect participant confidentiality.

### Funding

This work received no university external funding. The FamuPlus project was developed and implemented within the Institute of General Practice at Friedrich-Alexander-Universität Erlangen-Nürnberg as part of an educational development initiative.

### Author contributions

RK and BE share the last authorship.

AP and RK conceptualized the FamuPlus project and conducted focus group discussions. RK designed the online training. AP analysed the data and drafted the manuscript. TB and TK provided critical review and methodical guidance. SH contributed to the educational framework, manuscript editing and data management and analyzation. BE provided supervision, methodological guidance, and critical review and supported analysing the data. All authors agree to publication.

The present work was performed in partial fulfilment of the requirements for obtaining the degree “Dr. med.”

### Authors’ ORCIDs

- Andrea Punz: [0009-0004-2437-0265]
- Thomas Breakell: [0000-0003-1262-9124]
- Thomas Kuehlein: [0000-0003-2169-776X]
- Sigrid Harendza: [0000-0002-7920-8431]
- Raphael Kunisch: [0000-0002-0758-5721]
- Bettina Engel: [0000-0003-2660-5158]

## Acknowledgements

The authors wish to thank all participating students and clinical teachers for their time, engagement, and valuable feedback during the pilot phase of FamuPlus. 

## Competing interests

The authors declare that they have no competing interests. 

## Supplementary Material

Guideline for interview – second week of clinical elective

Guideline for focus group discussions

## Figures and Tables

**Table 1:**
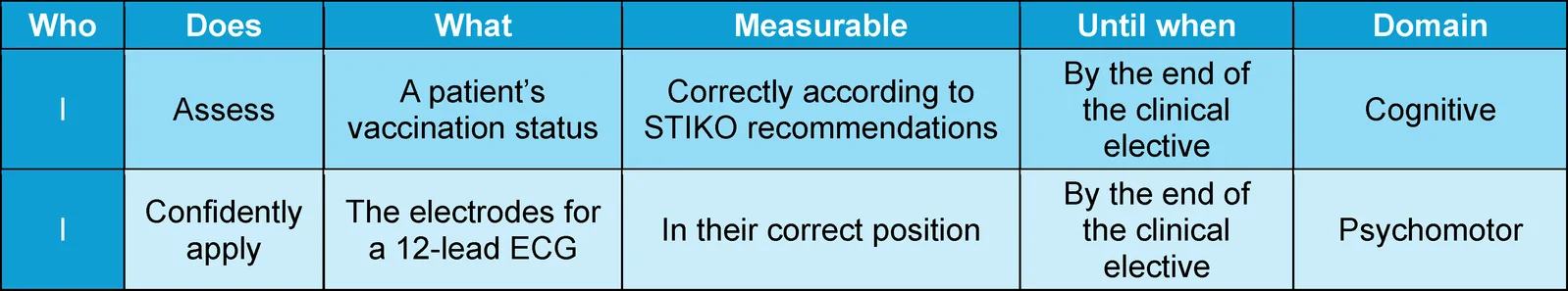
SMART template learning outcomes

**Table 2:**
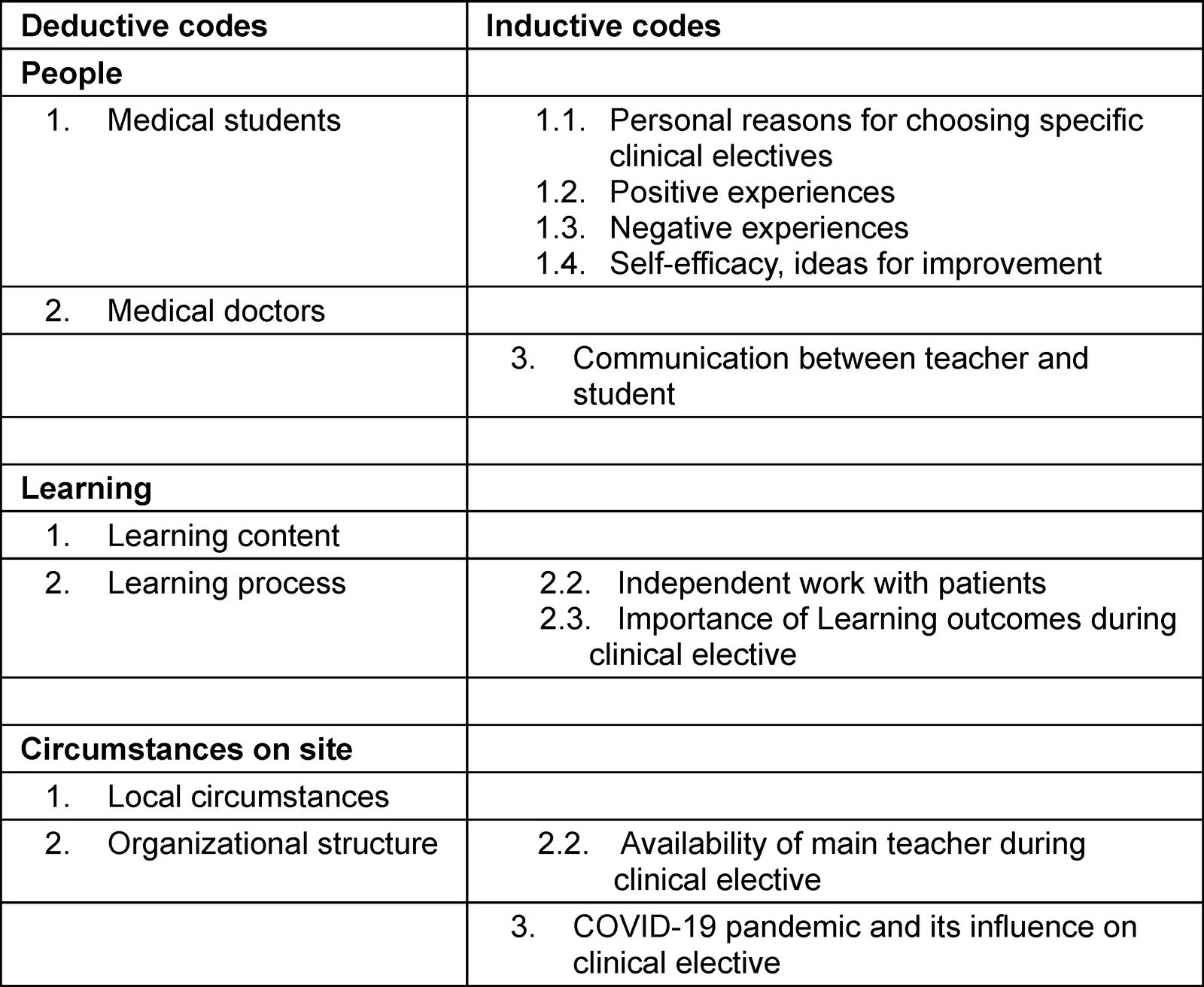
Deductive and inductive codes

**Table 3:**
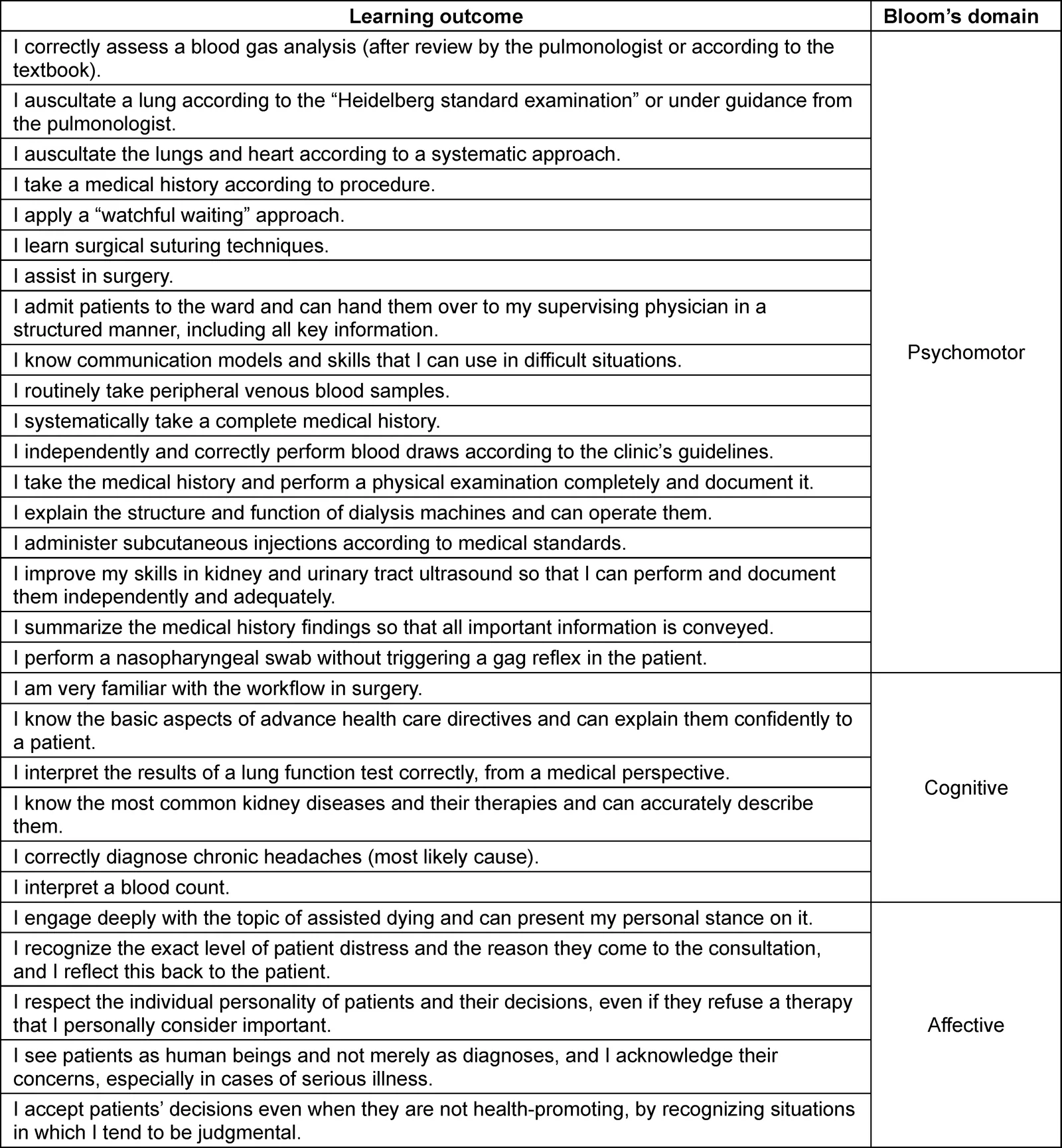
Learning outcomes of the seven students

**Figure 1:**
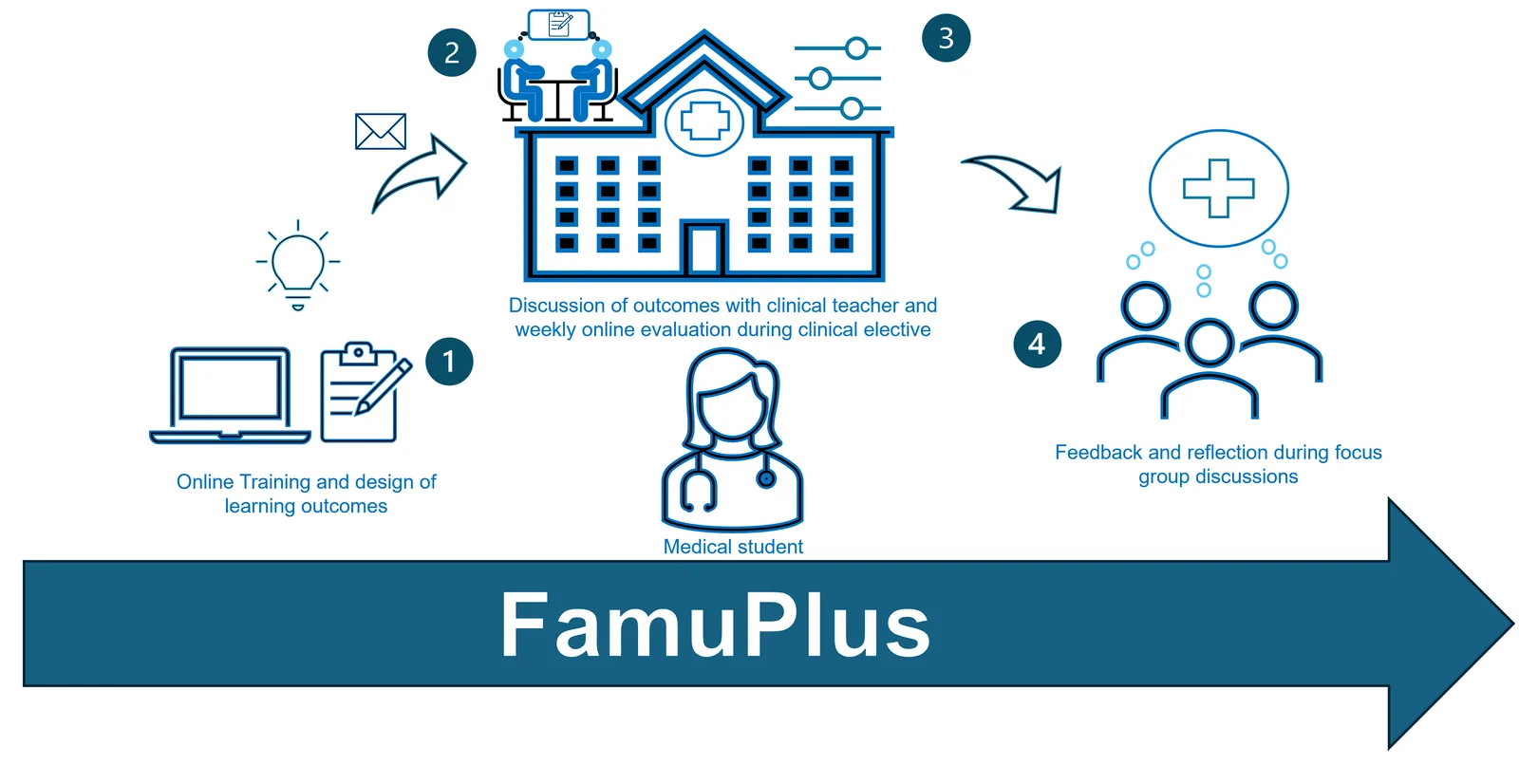
Overview of the FamuPlus schedule
