# Supplementary material for: FamuPlus – a pilot project to support self-oriented learning through self-created learning outcomes in clinical electives
Source: GMS J Med Educ. 2026 Sep 15;43(6):Doc76. doi: 10.3205/zma001870 (PMC13617270; doi:10.3205/zma001870)
Supplement: Supplementary file 1 [file JME-43-76-s-001.pdf]

Attachment 1: Guideline for interview – second week of clinical elective

Welcome; Information and consent to Recording of the interview and anonymization of data

1. How was the first week of your clinical election?
    a. What is going well?
    b. What would you wish to be different? What (of these) could you actively affect?
2. Did you discuss your learning objectives?
    a. If so: How did the meeting go?
    b. If not: Why not?
3. What role have your learning objectives played in your clinical elective so far?
4. What are your plans for the rest of your clinical elective?
5. Do you have any more questions? Is there anything else we can help you with?

Thanks and Goodbyes

Attachment 1 to Punz A, Breakell T, Kühlein T, Harendza S, Kunisch R, Engel E. *FamuPlus – a pilot project to support self-oriented learning through self-created learning outcomes in clinical electives.* GMS J Med Educ. 2026;43(6):Doc76. DOI: 10.3205/zma001870
