# Supplementary material for: FamuPlus – a pilot project to support self-oriented learning through self-created learning outcomes in clinical electives
Source: GMS J Med Educ. 2026 Sep 15;43(6):Doc76. doi: 10.3205/zma001870 (PMC13617270; doi:10.3205/zma001870)
Supplement: Supplementary file 2 [file JME-43-76-s-002.pdf]

Attachment 2: Guideline for focus group discussions

Welcome; Information and consent to Recording of the interview and anonymization of data

1. Why did you choose this placement? Why this discipline in this location?
2. How was your clinical election?
    a. What was going well?
    b. What would you wish had been different? What (of these) could you actively affect?
3. Did you have a designated contact person for questions? If yes: What was this person’s function (e.g. doctor, medical assistant, nurse?)
4. Did you discuss your learning objectives?
    a. If so: How did the meeting go?
    b. If not: Why not?
5. What role have your learning objectives played in your clinical elective?
6. What are your plans for your next clinical elective?
    a. What discipline?
    b. What are your wishes in terms of content?
    c. What do you expect from yourself?
    d. In relation to your learning objectives, should you have any: Would you do anything fundamentally different in the process of designing them?

Thanks and Goodbyes

Attachment 2 to Punz A, Breakell T, Kühlein T, Harendza S, Kunisch R, Engel E. *FamuPlus – a pilot project to support self-oriented learning through self-created learning outcomes in clinical electives.* GMS J Med Educ. 2026;43(6):Doc76. DOI: 10.3205/zma001870
